# *Pseudomonas* sp. G31 and *Azotobacter* sp. PBC2 Changed Structure of Bacterial Community and Modestly Promoted Growth of Oilseed Rape

**DOI:** 10.3390/ijms252313168

**Published:** 2024-12-07

**Authors:** Jakub Dobrzyński, Iryna Kulkova, Zuzanna Jakubowska, Aleksandra Naziębło, Barbara Wróbel

**Affiliations:** Institute of Technology and Life Sciences—National Research Institute, Falenty, 3 Hrabska Avenue, 05-090 Raszyn, Poland; i.kulkova@itp.edu.pl (I.K.); z.jakubowska@itp.edu.pl (Z.J.); a.nazieblo@itp.edu.pl (A.N.); b.wrobel@itp.edu.pl (B.W.)

**Keywords:** PGPB, native bacteria, plant growth stimulation

## Abstract

Oilseed rape is one of the most important oilseed crops, requiring high levels of nitrogen fertilization. Excessive nitrogen use, however, leads to numerous negative environmental impacts, spurring the search for sustainable, environmentally friendly alternatives to reduce reliance on mineral nitrogen fertilizers. One promising approach involves plant-growth-promoting bacteria (PGPB), which can support oilseed rape growth and lessen the need for traditional nitrogen fertilizers. This study evaluates a selected microbial consortium comprising bacterial isolates obtained from soil: *Pseudomonas* sp. G31 and *Azotobacter* sp. PBC2 (P1A). The applied PGPB significantly increased seed yield (a 27.12% increase) and, in the initial phase of the study, reduced the ammonium nitrogen content in the soil (a 20.18% decrease). Metataxonomic analyses were performed using Next-Generation Sequencing (NGS) technology by Illumina. Although P1A did not significantly affect alpha diversity, it altered the relative abundance of some dominant soil microorganisms. In the BBCH 75 phase, the P1A consortium increased the abundance of bacteria of *Firmicutes phylum*, including the genera *Bacillus* and *Paenibacillus*, which was considered a beneficial change. In summary, the *Pseudomonas* sp. G31 and *Azotobacter* sp. PBC2 consortium increased seed yield and was found to be part of the native rhizosphere community of oilseed rape, making it a promising candidate for commercialization.

## 1. Introduction

Oilseed rape (*Brassica napus* L.) is a vital crop in agriculture and environmental sustainability, ranking among the most significant oil crops after soybean and palm oil. It serves as a renewable source of high-quality vegetable oil, widely utilized in food production, biofuels, and various industrial applications. Additionally, oilseed rape contributes to sustainable farming by improving soil structure, acting as an effective break crop in rotations, and supporting biodiversity by providing important resources for pollinators [1,2]. According to a recent FAO report, the cultivated area for oilseed rape has steadily expanded over recent decades, reaching approximately 35 million hectares in 2023 and yielding around 69 million tons [3]. Within the European Union, France and Germany hold the largest oilseed rape cultivation areas, each producing approximately 4.2 million tons annually, while Poland ranks 10th in production [3]. Overall, oilseed rape accounts for 0.75% of the EU’s total agricultural area. Oilseed rape has high nitrogen requirements, making effective nitrogen fertilization management essential for optimal plant development and enhancing seed yield and seed oil content [4,5]. However, excessive nitrogen application to crops can lead to significant environmental issues, including nitrogen leaching into groundwater, the emission of greenhouse gases (e.g., N_2_O), and soil acidification [6,7,8,9,10,11].

A sustainable approach to reducing the use of mineral fertilizers, including nitrogen fertilizer, for crops is the application of plant-growth-promoting bacteria (PGPB). These bacteria possess various traits that can enhance both crop yield and quality [12,13]. Key bacterial metabolites that promote plant growth include indole-3-acetic acid (IAA), 1-aminocyclopropane-1-carboxylate (ACC) deaminase, nitrogenase (which enables atmospheric nitrogen fixation), and organic acids that solubilize forms of phosphorus otherwise unavailable to plants [14,15,16,17]. PGPB encompass a variety of bacterial genera, such as *Azospirillum* [18], *Pseudomonas* [19], *Bacillus* [20], *Paenibacillus* [17], *Azotobacter* [21], *Serratia* [22], and *Enterobacter* [23].

Despite extensive research on the impact of PGPB on crops [24], there is still a limited understanding of their impact on the native soil microbiota. It is particularly important to assess the post-application changes in the rhizosphere—the zone of highest microbial diversity and most intense plant–bacteria interactions [25]. However, research on PGPB’s effects on the native bacterial community in crops remains limited [26,27], particularly under field conditions. In particular, there have been few studies focusing on consortia containing bacteria of the genera *Pseudomonas* and *Azotobacter*, despite their potential to significantly stimulate the growth of oilseed rape [26,28]. Therefore, this study aims to evaluate the effects of a novel consortium of *Pseudomonas* sp. G31 and *Azotobacter* sp. PBC2 (P1A) not only on plant growth but also on the diversity and structure of the bacterial community at the taxonomic level in the rhizosphere of oilseed rape using Next-Generation Sequencing. Importantly, our metataxonomic analyses were conducted at three different plant growth stages, providing a comprehensive view of post-application changes in the native bacterial community.

## 2. Results and Discussion

### 2.1. Plant Growth-Promoting Effects

Approximately 200 bacterial strains were initially isolated, followed by preliminary studies to identify those exhibiting traits such as phytohormone production, atmospheric nitrogen fixation, and phosphorus solubilization. From this screening process, two bacterial strains were selected for further investigation, as they collectively displayed all of the desired PGP traits. The bacterial strains were identified through 16S rRNA gene sequencing, with the resulting contigs deposited in GenBank under the following accession numbers: *Pseudomonas* sp. G31—PP499654 and *Azotobacter* sp. PBC2—PP500530. In plate assays, the selected isolates did not exhibit antagonism towards each other. As noted, both strains exhibited plant-growth-promoting properties: *Pseudomonas* sp. G31 produced 95.4 µg mL^−1^ of indole-3-acetic acid (IAA) and demonstrated phosphate-solubilizing abilities, while *Azotobacter* sp. PBC2 produced 43.6 µg mL^−1^ of IAA and could fix atmospheric nitrogen.

Soil inoculation with the bacterial consortium P1A did not significantly affect either the growth of shoots and roots or biomass (Table 1). While there was only a minor increase in the dry weight of P1A-treated plants, the seed yield showed a significant enhancement, increasing by over 27% in P1A-inoculated plants.

Minuț et al. [29] tested the plant-growth-promoting (PGP) properties of three bacterial strains, including *Pseudomonas* sp. and *Azotobacter* sp., on several crop species and obtained similar results: none of the strains stimulated oilseed rape root growth, and some even reduced the root weight. The impact on shoot growth varied considerably on native soil microorganisms, but dry mass was not significantly affected by the treatments [29]. However, the lack of observed growth stimulation does not rule out positive inoculation effects on other parameters, such as chlorophyll content or resistance to biotic and abiotic stresses [30]. Świątczak et al. [30] evaluated the ability of five *Pseudomonas* species to promote oilseed rape growth and development. They observed that most strains did not affect the root and shoot length or biomass; however, all strains produced IAA and siderophores, with some also demonstrating chitinase production, ACC deaminase activity, and phosphate-solubilization capabilities [30]. It is possible that mutual inhibition among bacterial strains may limit beneficial PGP effects. Similarly, Hassouna et al. [31] reported a significant increase in onion bulb growth and macronutrient accumulation following inoculation with *Pseudomonas* sp., *Azotobacter* sp., and *Azospirillum* sp. strains, although a mixed inoculant was notably less effective for plant growth promotion than single-strain applications.

The increase in seed yield observed in our study confirms that the P1A consortium possesses PGP properties, even though these are not reflected in the stimulation of root and shoot growth. This outcome is highly promising for agricultural applications.

### 2.2. Physico-Chemical Analysis of Rhizosphere Soil

No notable changes in the physico-chemical properties of rhizosphere soil were observed following P1A inoculation (Table 2, Supplement Table S1). Soil pH remained stable across treatments, with values being consistently acidic, ranging from 4.73 to 4.87. The levels of total organic carbon (TOC) and total nitrogen (TN) content also remained unchanged, although slight treatment-related differences were observed in the ammonia and nitrate forms of nitrogen. Both N-NO_3_ and N-NH_4_ content decreased at the first time point, with only ammonia showing a statistically significant reduction of approximately 20%. Differences in these nitrogen forms at the subsequent time points were minimal. This result is somewhat surprising, given that *Azotobacter* species are diazotrophic and generally enrich soil with available nitrogen [32,33]. It is possible that early-season conditions—such as the temperature, oxygen availability, and inorganic compound presence—were suboptimal for nitrogen fixation [34].

P1A inoculation appeared to increase the available phosphorus (AP) content, a result anticipated due to the phosphate-solubilizing activity of *Pseudomonas* sp. strain G31, though the change was not statistically significant. Some studies report significant increases in nutrient uptake following rhizosphere inoculation with *Azotobacter* and *Pseudomonas* strains. Sharafzadeh [35] noted elevated levels of calcium and magnesium in tomatoes, while Sultana et al. [36] reported higher iron accumulation in sorghum. Although we did not analyze the nutrient concentrations in oilseed rape, it is possible that the increase in seed yield was a result of intensified macro- and microelement uptake.

### 2.3. Bacterial Community

#### 2.3.1. Alpha Diversity

The bacterial community’s response to the consortium application was assessed by sequencing the V3–V4 regions of the 16S rRNA genes, resulting in 1,646,071 raw sequences. Bioinformatic analysis of the soil samples yielded the following ranges for alpha-diversity parameters: Chao 1559–2902, observed features 1545–2830, Shannon 9.21–10.14, and Simpson 0.90–0.99 (Table 3, Supplement Table S2). Statistical analysis of these parameters across time points showed no significant differences between PGPB-inoculated samples and the control samples. Bacterial diversity in soil is essential for maintaining soil health and supporting ecosystem functions. A diverse bacterial community contributes to efficient nutrient cycling, the decomposition of organic matter, and the overall stability and resilience of soil ecosystems [37,38]. Thus, the lack of impact of PGPB on alpha diversity is generally favorable and consistent with several previous studies. For instance, after applying *Arthrobacter* and *Microbacterium* in controlled conditions, Samain et al. [39] reported no differences in the alpha diversity of the bacterial community between an inoculated wheat rhizosphere and control. Similarly, Lee et al. [40] found no significant alpha diversity changes in field-grown-tomato soil after applying *Rhodopseudomonas palustris* PS3. On the other hand, some studies have observed a significantly increased alpha diversity following PGPB application. For instance, a consortium of *Acinetobacter beijerinckii* LJL-12 and *Pseudomonas fluorescens* MJM-5 significantly increased the alpha diversity in an alfalfa rhizosphere under field conditions [41]. Such variations across studies may result from differences in PGPB strains, soil types, plant species, and climatic conditions.

#### 2.3.2. Beta Diversity

**Figure 1:**
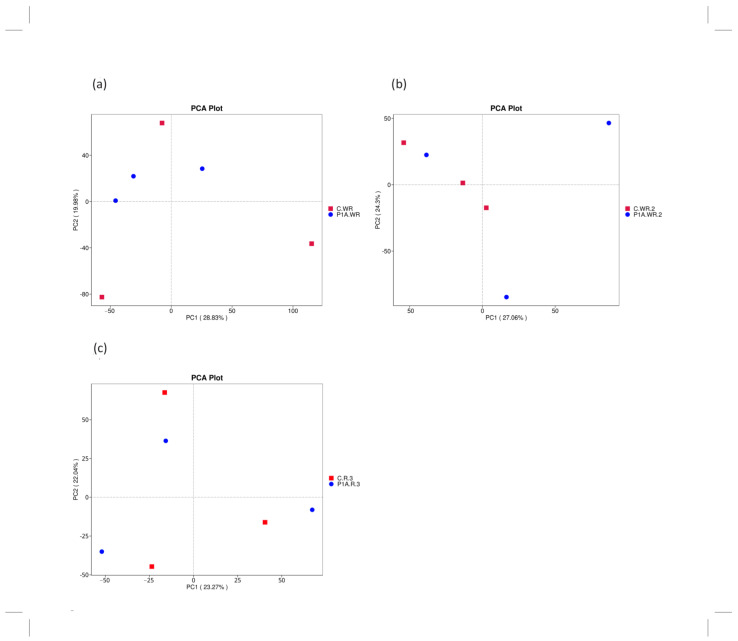
PCA of the rhizobacterial community: C.R—control rhizosphere, P1A.R—rhizosphere inoculated with P1A; (**a**)—first time point, (**b**)—second time point, (**c**)—third time point.

Overall, the samples exhibited diversity at each time point, as indicated by the relatively low values on the PC1 and PC2 axes (Figure 1). At the first time point, all samples from the inoculated P1A soil were relatively similar to one another, whereas control samples showed greater variability along PC1 (19.98%) and PC2 (28.83%) (Figure 1a). At the second time point, the control samples formed a pair, while the PGPB-inoculated samples showed distinct separation along both axes (Figure 1b). In the samples collected before harvest, no clear pattern of similarity was observed on the plot (Figure 1c). This moderate differentiation of bacterial communities may reflect soil variability across experimental plots and differences in the rhizodeposit profiles, such as root exudates. Moderate sample heterogeneity within treatments (both control and PGPB-inoculated samples) was similarly observed following PGPB application in the cultivation of durum wheat, where non-metric multidimensional scaling (NMDS) was used [42]. Comparable results were also reported by Wang et al. [43], who studied phosphate-solubilizing bacteria in the peanut field.

#### 2.3.3. Structure of Bacterial Communities

**Figure 2:**
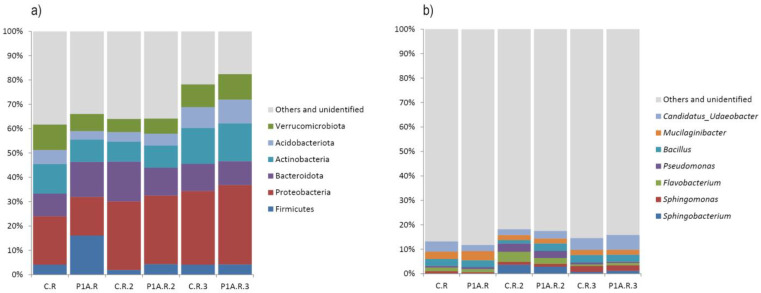
Relative abundance of the dominant bacterial phyla (**a**) and genera (**b**): C.R—control rhizosphere, P1A.R—rhizosphere inoculated with P1A; 1—first time point, 2—second time point, 3—third time point.

**Figure 3:**
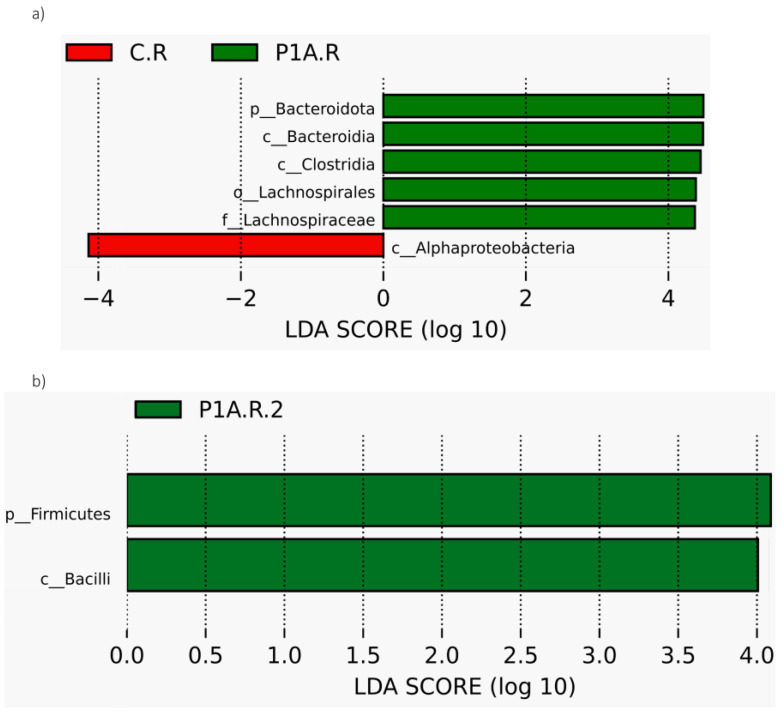
LEfSe analysis of the rhizobacterial community: C.R—control rhizosphere P1A.R—inoculated with P1A; (**a**)—first time point, (**b**)—second time point; there were no significant differences at the third time point.

**Figure 4:**
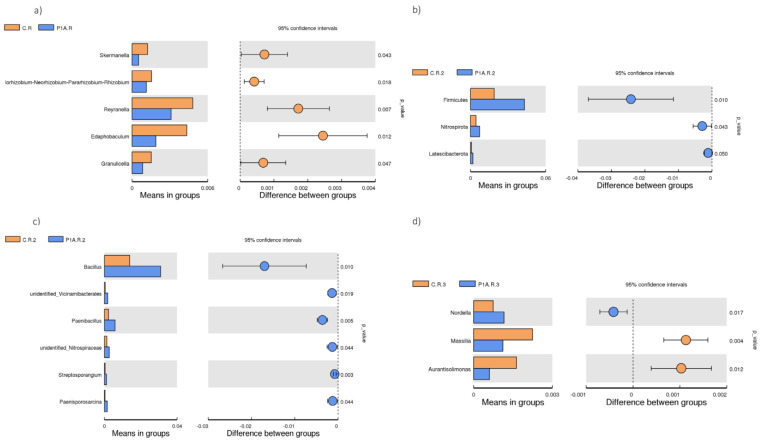
A *t*-test of the rhizobacterial community: C.R—control rhizosphere, P1A.R—inoculated with P1A; phyla—(**b**) (three time points); genera—(**a**,**c**,**d**) (three time points).

In the analyzed samples, several bacterial taxa were dominant, including Proteobacteria (32.7–15.9%), Actinobacteriota (15.5–8.2%), Acidobacteriota (9.8–3.5%), Bacteroidota (16.4–9.3%), Firmicutes (16.1–1.9%), and Verrucomicrobiota (10.5–5.4%) (Figure 2a). The LefSe test (Figure 3a) indicated a significantly higher presence of the Bacteroidota in the P1A-inoculated soil during the first sampling period; however, this result was not confirmed by the *t*-test and MetaStat. In the second sampling period, both the LefSe test and the *t*-test, as well as MetaStat (Figure 3b, Figure 4b, and Figure S1), showed a significantly higher abundance of Firmicutes in the inoculated samples compared to the control. Additionally, the *t*-test and MetaStat indicated a significantly higher abundance of non-dominant taxa, specifically Nitrospirota and Latescibacterota, in the inoculated soil. By the third sampling period, no significant differences in the bacterial community of the studied soil were observed at the phylum level.

The dominant genera in the studied soil samples included *Candidatus*_Udaeobacter (6.1–2.4%), *Flavobacterium* (4.1–0.7%), *Mucilaginibacter* (3.8–2%), *Sphingobacterium* (3.6–0%), *Sphingomonas* (2.4–0.6%), *Pseudomonas* (3.4–0.5%), and *Bacillus* (3.1–1.4%) (Figure 2b). No significant shifts in the bacterial community at the genus level were observed three weeks post-inoculation. However, changes were detected among non-dominant taxa, with the *t*-test and MetaStat revealing a significantly higher abundance of several Alpha-proteobacteria genera—*Skermanella*, *Allorhizobium-Neorhizobium-Pararhizobium-Rhizobium*, and *Reyranella*—as well as *Edaphobaculum* and *Granulicella* (both from the Bacteroidota phylum) in control samples compared to inoculated ones (Figure 4a and Figure S1). Due to the low relative abundances of these taxa, these changes are unlikely to be detrimental. Importantly, the LefSe test did not reveal significant differences. In the second sampling period, the consortium-inoculated soil showed a notable increase in Bacillus (Firmicutes phylum) among the dominant taxa (Figure 4c), although LefSe confirmed only an increase in the Bacilli class (Figure 3b). Additionally, an increased abundance of certain non-dominant bacteria was noted in the inoculated soil, including *Paenibacillus* (Firmicutes phylum), Unidentified Vicinamibacterales (Acidobacteriota phylum), Unidentified Nitrospiraceae (Nitrospirota phylum), Streptosporangium (Actinobacteriota phylum), and *Paenisporosarcina* (Firmicutes phylum) (Figure 4c and Figure S1). In samples collected before harvest, the *t*-test and Metastat revealed only three changes in non-dominant taxa: a significantly higher abundance of *Massilia* and *Aurantisolimonas* in non-inoculated PGBP samples compared to the control rhizosphere, while *Nordella* was significantly more abundant in the inoculated soil (Figure 4d and Figure S1). Given the low dominance of these taxa, their role in soil biochemical processes or interactions with the plant are likely minimal. Additionally, the LefSe test did not detect any shifts in the rhizobacterial community at the last time point.

Considering the results, the most notable post-application change in the dominant taxa is the increased abundance of Firmicutes in the second sampling point, confirmed by all statistical tests. This increase in Firmicutes was primarily manifested by the growth of *Bacillus*, a dominant genus, alongside a rise in non-dominant *Paenibacillus*. This shift appears beneficial, as the bacteria from the *Bacillus* and related genera, such as *Paenibacillus*, produce enzymes critical for soil nutrient cycling, including cellulases, chitinases, amylases, and glucanases [17,44,45,46]. Moreover, these taxa can stimulate plant growth by secreting IAA and cytokinins, producing nitrogenase, or solubilizing phosphorus [47,48,49,50]. They may also enhance plant health by controlling phytopathogens via antibiotic production and triggering Induced Systemic Resistance (ISR) [17].

However, a moderately important shift in the inoculated bacterial community, confirmed by two statistical tests, was observed as a decrease in the abundance of certain genera within the class Alpha-proteobacteria, including *Skermanella*, *Allorhizobium-Neorhizobium-Pararhizobium-Rhizobium*, and *Reyranella.* As was noted earlier, these genera are not among the dominant bacteria in the soil, suggesting that this change is unlikely to have a substantial negative impact on the soil’s biochemical processes. Despite their lower abundance, these genera play notable roles in the soil ecosystem. For instance, *Allorhizobium-Neorhizobium-Pararhizobium-Rhizobium* species can form symbiotic relationships with legumes [51]. They can also utilize some carbohydrates and nitrogen sources, contributing to element cycling in the soil [52,53]. Additionally, some members of *Skermanella* possess the ability to degrade starch [54].

Alterations in bacterial communities following PGPB inoculation have been documented in various studies, both under controlled conditions and in field experiments [26,43,55]. However, there is still limited research on the impact of PGPB on the soil microbiota in oilseed rape cultivation, particularly studies involving *Azotobacter* spp. and *Pseudomonas* spp. in field experiments with oilseed rape. For instance, our previous study [28] demonstrated that introducing a different consortium into oilseed rape cultivation, although composed of members from the same genera, elicited a distinct response in the rhizobacterial community. Three weeks after the inoculation with *Pseudomonas* sp. KR227 and *Azotobacter* sp. PBC1, there was an observed increase in the relative abundance of Proteobacteria, a pattern that differed from the current study. Additionally, a reduction in the abundance of Verrucomicrobiota was noted. 

Liu et al. [26] also detected changes in the rhizosphere community structure of oilseed rape (greenhouse study) after applying three strains: *Stenotrophomonas rhizophila*, *Rhodobacter sphaeroides*, and *Bacillus amyloliquefaciens*. They found that *R. sphaeroides* and *B. amyloliquefaciens* selectively enhanced the growth of *Pseudomonadaceae* and *Flavobacteriaceae* members, while *S. rhizophila* increased populations of Cyanobacteria and Actinobacteriota [26]. Similarly, in a cucumber rhizosphere study, *B. amyloliquefaciens* FH-1 significantly boosted the population of the Deltaproteobacteria class (*Proteobacteria phylum*) in a pot experiment [55]. Interestingly, the introduction of the same strain in lettuce cultivation led to a decrease in the relative abundance of Proteobacteria after two weeks [56]. These discrepancies between studies may stem not only from the use of different PGPB strains but also from variations in plant species, soil properties, or cultivation conditions.

## 3. Materials and Methods

### 3.1. Plant-Growth-Promoting Effects

#### 3.1.1. Isolation, Selection, and Taxonomic Identification of PGPB

In this study, approximately 200 bacterial strains were isolated from various soils. Rhizosphere and bulk soils were diluted to concentrations of 10^−6^. Subsequently, 1 mL of the diluted suspension was inoculated onto nutrient agar plates. Colonies were selected and subjected to repeated passaging until pure bacterial strains were obtained.

The bacterial strains were identified through 16S rRNA gene sequencing. Genomic DNA was extracted from bacterial strains cultivated for 16 h using the Genomic Mini kit (A&A Biotechnology, Gdansk, Poland). The universal primers, 27F (5′-AGAGTTTGATCCTGGCTCAG-3′) and 1492R (5′-GGTTACCTTGTTACGACTT-3′), were utilized to amplify the 16S rRNA genes. PCR amplification was conducted under the following conditions: an initial denaturation at 95 °C for 3 min; 30 cycles of denaturation at 95 °C for 30 s, annealing at 55 °C for 2 min, and extension at 72 °C for 2 min; this was followed by a final incubation at 72 °C for 10 min to complete DNA amplification. The PCR products were sequenced using the Sanger method (NEXBIO, Lublin, Poland). Forward and reverse reads were assembled into contigs using BioEdit (version 7.2) and compared against sequences from the GenBank and EMBL databases via BLAST (Basic Local Alignment Search Tool). The 16S rRNA gene sequences of the studied bacterial strains have been deposited in GenBank with the following accession numbers: *Pseudomonas* sp. G31—PP499654 and *Azotobacter* sp. PBC2—PP500530.

#### 3.1.2. IAA Production

The production of indole-3-acetic acid (IAA) was assessed using the method described by Bric et al. [57]. For this assessment, bacterial isolates were cultured in Luria–Bertani (LB) broth supplemented with 1000 μg L^−1^ tryptophan and incubated for 72 h at 30 ± 2 °C. After centrifugation at 12,000 rpm for 10 min (4 °C), 1 mL of supernatant was mixed with 2 mL of Salkowski reagent (49 mL of 35% perchloric acid and 1 mL of 0.5 M FeCl_3_ solution). Then, the mixture was incubated in the dark at room temperature for 90 min. Subsequently, absorbance was measured at 530 nm using a spectrophotometer (Epoch 2 Plate Reader, BioTek, Shoreline, WA, USA). Bacterial IAA production was quantified by comparison to a standard IAA curve calibrated in the range of 10–100 μg mL^−1^.

#### 3.1.3. Nitrogen Fixation

A nitrogen fixation assay for the bacterial strains was conducted using nitrogen-free Burk’s medium, which contained the following components: MgSO_4_ (0.2 g), K_2_HPO_4_ (0.8 g), KH_2_PO_4_ (0.2 g), CaSO_4_ (0.13 g), FeCl_3_ (0.001 g), NaMoO_4_ (0.0003 g), and sucrose (20 g), supplemented with 18 g L^−1^ of agar. The cultures were incubated at 30 °C for 7 days. Bacteria that exhibited growth on this medium were identified as diazotrophs.

#### 3.1.4. Phosphate Solubilization

Bacterial strains were spot-transferred onto Pikovskaya agar, prepared per liter with the following components: yeast extract 0.5 g, dextrose 10.0 g, Ca_3_(PO_4_)_2_ 5.0 g, (NH_4_)_2_SO_4_ 0.5 g, KCl 0.2 g, MgSO_4_ 0.1 g, MnSO_4_ 0.0001 g, FeSO_4_ 0.0001 g, and 15.0 g of agar, supplemented with 2% tricalcium phosphate (TCP). The cultures were incubated at 30 ± 2 °C for 94 h. Phosphorus solubilization ability was assessed by observing the formation of clear zones around bacterial colonies.

#### 3.1.5. Experimental Treatments and Sampling Procedures

The research was conducted in a field owned by Awista Pierwsza in Kobierzycko (Sieradz Structural Basin, Southern Greater Poland Lowland), Sieradz district, Łódź Voivodeship (51°37′59.2″ N, 18°36′50.9″ E). Winter oilseed rape (*Brassica napus* L.; variety Absolut) was sown at a density of 40 seeds per m^2^ in August 2022 on Luvisol clay soil. In autumn 2022, the field was fertilized with Macro Speed Optima (16% P, 32% K, 11% Ca, 8% SO_3_, 8% Zn) at a rate of 200 kg ha^−1^ and urea with a urease inhibitor (46% N) at 180 kg ha^−1^. The average soil pH at the beginning of the growing season was measured at 6.1.

During the experiment, the average monthly temperature and precipitation were as follows: April (24.9 mm, 7.8 °C), May (19 mm, 13.2 °C), June (30.5 mm, 18.2 °C), and July (85.1 mm, 20.0 °C). In spring 2023, a one-factor field experiment was established on the production field using a randomized block design with three replicates. Each plot measured 2 m × 5 m (10 m^2^), with a meter-wide buffer zone between plots.

The experiment included two treatments: a control group (C) and a treatment with a bacterial consortium consisting of *Pseudomonas* sp. G31 and *Azotobacter* sp. PBC2, designated as P1A. The bacterial culture, established to prepare the suspension, was centrifuged at 3000 rpm, and then an inoculum with a McFarland scale value of 0.5 was prepared from the bacterial cells, corresponding to approximately 1.5 × 10^8^ CFU mL^−1^ (densitometer Biosan DEN-1). The inoculum was mixed in a 1:1 ratio. The bacterial consortium was applied in April 2023 (BBCH 35), using a hand sprayer to apply 600 mL of inoculum per plot.

Rhizosphere soil samples were collected three times during the growing season: three weeks post-application (at the oilseed rape development stage BBCH 65), mid-season (BBCH 75), and at harvest (BBCH 90). Samples were collected from three plots for each treatment (e.g., three from C.R.), with three plants sampled per plot to obtain rhizosphere soil. To isolate the rhizosphere, loosely bound soil was gently shaken off, while tightly bound soil was removed using a sterile brush, then sieved through a sterile sieve [58]. Samples were stored at −20 °C for chemical analyses and at −80 °C for metataxonomic analysis. Before harvesting, data were collected from 30 plants in each plot, including measurements of plant height, the number of shoots, root weight, and seed yield.

### 3.2. Physico-Chemical Analysis of Rhizosphere Soil

Rhizosphere soil samples were air-dried and sieved through a 2 mm mesh to determine pH, total carbon (TC), total nitrogen (TN), and available phosphorus (AP). Concentrations of N-NO_3_ and N-NH_4_ were measured in the fresh soil mass. The following methods were used to assess each parameter: pH was measured in 1 M KCl following PN-EN ISO 10390:2022-09 [59]; TC was determined according to the Polish standard PN-R-04024:1997 [60]; total TN was assessed following ISO 13878:1998 [61];, the concentrations of N-NH_4_ were evaluated using Continuous Flow Analysis (CFA) with spectrophotometric detection; and AP was measured following PN-R-04023:1996 [62].

### 3.3. Bacterial Community

Soil DNA was extracted using the Magnetic Soil and Stool DNA Kit (TianGen, Beijing, China). PCR amplification of the target regions was performed using specific primers for the V3–V4 region: 314F (5′-CCTACGGNGGCWGCAG-3′) and 785R (5′-GACTACHVGGTATCTAATCC-3′). Each PCR reaction contained 15 μL of Phusion^®^ High-Fidelity PCR Master Mix, 0.2 μM of forward and reverse primers, and approximately 10 ng of template DNA. Thermocycling conditions were as follows: initial denaturation at 98 °C for 1 min, followed by 30 cycles of denaturation at 98 °C for 10 s, annealing at 50 °C for 30 s, and elongation at 72 °C for 30 s, with a final extension at 72 °C for 5 min. The PCR products of the appropriate size were selected through electrophoresis on a 2% agarose gel.

Library preparation was completed with the NEB Next^®^ Ultra™ II FS DNA PCR-free Library Prep Kit (New England Biolabs, Ipswich, MA, USA), following the manufacturer’s protocol, and sequencing was performed on an Illumina NovaSeq 6000 System sequencer (Novogene, Germany), generating paired-end reads with 250 bp raw reads. The raw data were filtered and de-duplicated using the DADA2 method [63]. After noise reduction, the de-duplicated sequences were referred to as Amplicon Sequence Variants (ASVs). Taxonomic annotation was conducted using the Naive Bayes classifier [64] with the Silva 138.1 database. Based on the results of taxonomic annotation, the six most abundant phyla and seven most abundant genera (dominant taxa) were selected to construct a histogram displaying relative abundance. Dominant taxa at the phylum level were defined as those with a percentage abundance exceeding 4% in at least two treatments, while at the genus level, dominance was determined by a percentage abundance exceeding 1% in at least two treatments. To assess the diversity and richness of the samples, alpha diversity was evaluated using four indices (observed features, Chao1, Shannon, and Simpson) in QIIME2 (version 2023.5). Principal Component Analysis (PCA) and Principal Coordinate Analysis (PCoA) based on weighted UniFrac distances were conducted using the ade4 (version 1.7-22) and ggplot2 (version 3.4.2) packages in R (version 4.0.3).

Differences in the bacterial community at the phylum and genus levels were identified using LEfSe, *t*-tests, and MetaStat analyses with the vegan (version 2.6-4) and ggplot2 packages in R. Tukey’s HSD (Honest Significant Difference) test was applied with a significance threshold of *p* = 0.05. Statistical processing of the results was carried out using Statistica 6.0.

## 4. Conclusions

In conclusion, this field study demonstrated that a plant-growth-promoting bacteria (PGPB) consortium comprising *Pseudomonas* sp. G31 and *Azotobacter* sp. PBC2 significantly enhanced oilseed rape yield. At the BBCH 75 growth stage, the consortium also increased the relative abundance of Firmicutes, particularly the beneficial genera *Bacillus* and *Paenibacillus*, which may have further contributed to improving plant growth conditions. To gain a deeper understanding of the underlying mechanisms, future research should investigate the impact of the P1A consortium on soil microbial gene expression. This could involve examining how the consortium influences microbial genes related to nutrient cycling (e.g., genes involved organic matter decomposition), the plant stress response (e.g., genes associated with ISR), and plant–microbe interactions (e.g., genes involved in biofilm formation). Finally, expanding the research to other major crops with high nitrogen and nutrient requirements would also be valuable.

## Figures and Tables

**Table 1:** Plant growth traits after application of P1A; C.R—control rhizosphere, P1A.R—rhizosphere inoculated with P1A.

Treatment	Shoot Length (cm)	Shoot Weight (g)	Root Length (cm)	Root Weight (g)	Seed Yield (g)
**C.R**	157.5 a	1573.7 a	18.7 a	101.2 a	415.8 a
**P1A.R**	157 a	1773.3 a	18.4 a	114 a	528.6 b

Means in columns with the same letter do not differ significantly at *p* < 0.05 in Tukey’s HSD test.

**Table 2:** Physico-chemical properties of rhizosphere soil; C.R—control rhizosphere, P1A.R—rhizosphere inoculated with P1A; 1—first time point, 2—second time point, 3—third time point.

	1st Time Point		2nd Time Point		3rd Time Point	
**Treatment**	C.R	P1A.R	C.R.2	P1A.R.2	C.R.3	P1A.R.3
**pH**	4.87 a	4.83 a	4.8 a	4.73 a	4.83 a	4.87 a
**N-NO_3_ (mg kg^−1^)**	6.1 a	3.8 a	5.3 a	4.9 a	13.9 a	15.1 a
**N-NH_4_ (mg kg^−1^)**	8.77 b	7.0 a	11.67 a	12.27 a	9.87 a	10.3 a
**AP (mg kg^−1^)**	10.17 a	10.4 a	9.27 a	9.53 a	10.1 a	10.27 a
**TOC (%)**	1.42 a	1.42 a	1.45 a	1.42 a	1.43 a	1.43 a
**TN (%)**	0.127 a	0.12 a	0.14 a	0.127 a	0.14 a	0.13 a

AP—available P (P_2_O_5_); TOC—total organic carbon; TN—total nitrogen; means in rows with the same letter do not differ significantly at *p* < 0.05 in Tukey’s HSD test.

**Table 3:** Alpha diversity of oilseed rape bacterial community of rhizospheres. C.R—control rhizosphere, P1A.R—rhizosphere inoculated with P1A; 1—first time point, 2—second time point, 3—third time point.

	1st Time Point		2nd Time Point		3rd Time Point	
**Treatment**	C.R	P1A.R	C.R.2	P1A.R.2	C.R.3	P1A.R.3
**Chao1**	2902 a	2279 a	1650 a	1559 a	2067 a	2150 a
**Observed features**	2830 a	2221 a	1644 a	1545 a	2000 a	2067 a
**Shannon**	10.14 a	9.21 a	9.28 a	9.44 a	9.66 a	9.86 a
**Simpson**	0.995 a	0.990 a	0.992 a	0.996 a	0.993 a	0.996 a

Means in columns with the same letter do not differ significantly at *p* < 0.05 in Tukey’s HSD test.

## Data Availability

Sequencing data has been submitted to the Sequence Read Archive (SRA) at NCBI and is available under the accession number PRJNA1092498.

## Supplementary Materials

The supporting information can be downloaded at: https://www.mdpi.com/article/10.3390/ijms252313168/s1.
