# Supplementary material for: Pseudomonas sp. G31 and Azotobacter sp. PBC2 Changed Structure of Bacterial Community and Modestly Promoted Growth of Oilseed Rape
Source: Int J Mol Sci. 2024 Dec 7;25(23):13168. doi: 10.3390/ijms252313168 (PMC11642319; doi:10.3390/ijms252313168)
Supplement: Supplementary file 1 [file ijms-25-13168-s001.zip › ijms-3333016-supplementary.pdf]

Article

Pseudomonas sp. G31 and *Azotobacter* sp. PBC2 changed structure of bacterial community and modestly promoted growth of oilseed rape

Jakub Dobrzyński [1]*. Iryna Kulkova[1] . Zuzanna Jakubowska[1]. Aleksandra Naziębło[1] and Barbara Wróbel[1].

1 Institute of Technology and Life Sciences—National Research Institute. Falenty. 3 Hrabska Avenue. 05-090 Raszyn. Poland; j.dobrzynski@itp.edu.pl; i.kulkova@itp.edu.pl. z.jakubowska@itp.edu.pl. a.nazieblo@itp.edu.pl. b.wrobel@itp.edu.pl

* Correspondence: j.dobrzynski@itp.edu.pl;

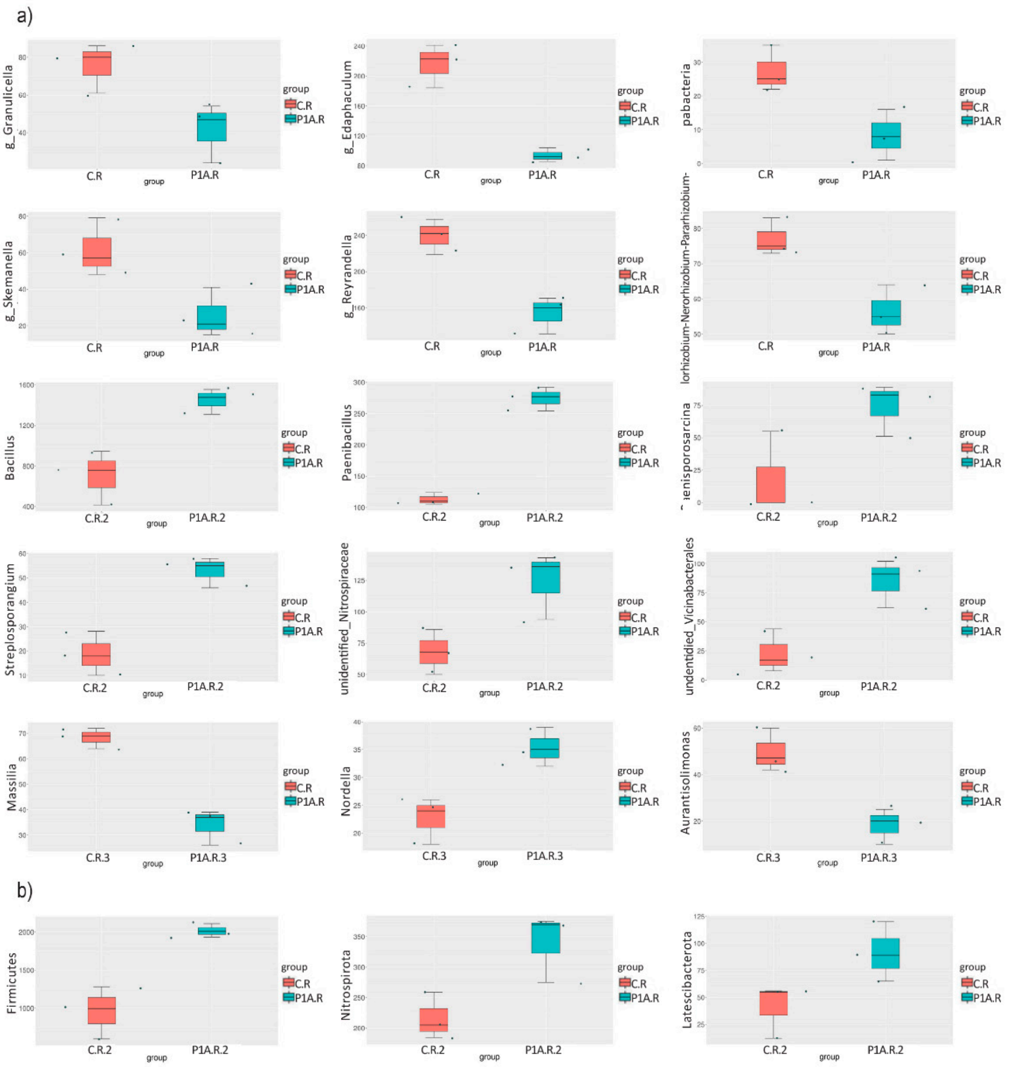

Supplement. Figure S1. MetaStat assay of bacterial community at genus level (a) and phylum level (b). C.R - control rhizosphere soil. P1A.R – rhizosphere inoculated with P1A; 1– first time point. 2–second time point. 3 – third time point.

Supplement. Table S1. Data of physico-chemical properties: C.R.– control rhizosphere – first time point (three repetitions) . P1A.R. – rhizosphere inoculated with P1A – first time point (three repetitions) etc.

Samples	pH w KCl	N-NO3	N-NH4	available P	TOC (%)	TN (%)
C1.R	4.7	6.2	8.50	8.30	1.41	0.13
C2.R	5	6.1	9.10	11.80	1.46	0.13
C3.R	4.9	5.9	8.70	10.40	1.40	0.12
C.R	4.87	6.1	8.77	10.17	1.42	0.13
P1A1.R	4.70	2.1	7.60	7.60	1.29	0.11
P1A2.R	5.00	5.5	7.00	13.70	1.44	0.13
P1A3.R	4.80	3.8	6.50	9.90	1.54	0.12
P1A.R	4.83	3.8	7.03	10.40	1.42	0.12
C1.R.2	4.80	3.2	13.20	11.30	1.53	0.13
C2.R.2	4.80	5.3	9.90	8.50	1.55	0.14
C3.R.2	4.80	7.3	11.90	8.00	1.27	0.14
C.R.2	4.80	5.3	11.67	9.27	1.45	0.14
P1A1.R.2	4.60	5.1	12.70	9.30	1.41	0.12
P1A2.R.2	4.80	5.7	11.80	11.10	1.41	0.13
P1A3.R.2	4.80	3.9	12.30	8.20	1.44	0.13
P1A.R.2	4.73	4.9	12.27	9.53	1.42	0.13
C1.R.3	4.90	14.9	9.20	9.90	1.44	0.14
C2.R.3	4.70	12.2	8.30	10.40	1.51	0.13
C3.R.3	4.90	14.6	12.10	10.00	1.35	0.15
C.R.3	4.83	13.9	9.87	10.10	1.43	0.14
P1A1.R.3	4.80	13.2	8.90	10.10	1.49	0.13
P1A2.R.3	4.80	16.3	10.70	8.50	1.47	0.13
P1A3.R.3	5.00	15.8	11.30	12.20	1.34	0.13
P1A.R.3	4.87	15.1	10.30	10.27	1.43	0.13

Supplement. Table S2. Data of alfa-diversity: C.R.1– control rhizosphere – first time point (three repetitions) . P1A.R – rhizosphere inoculated with P1A – first time point (three repetitions) etc.

Samples	chao1	dominance	goods_coverage	observed_features	pielou_e	shannon	simpson
C1.R	2946.48	0.00	1.00	2864.00	0.90	10.36	1.00
C2.R	3055.39	0.00	1.00	2985.00	0.89	10.32	1.00
C3.R	2705.89	0.01	1.00	2640.00	0.86	9.74	0.99
C.R	2902.59	0.01	1.00	2829.67	0.88	10.14	1.00
P1A1.R	2590.34	0.01	1.00	2531.00	0.87	9.81	1.00
P1A2.R	2689.20	0.01	1.00	2616.00	0.85	9.61	0.99
P1A3.R	1558.66	0.01	1.00	1516.00	0.78	8.22	0.99
P1A.R	2279.40	0.01	1.00	2221.00	0.83	9.21	0.99
C1.R.2	2128.88	0.00	1.00	2122.00	0.91	10.00	1.00
C2.R.2	1359.80	0.01	1.00	1354.00	0.84	8.76	0.99
C3.R.2	1460.64	0.01	1.00	1455.00	0.87	9.10	0.99
C.R.2	1649.77	0.01	1.00	1643.67	0.87	9.29	0.99
P1A1.R.2	1612.40	0.00	1.00	1602.00	0.91	9.71	1.00
P1A2.R.2	1412.38	0.01	1.00	1396.00	0.86	9.01	0.99
P1A3.R.2	1652.65	0.00	1.00	1638.00	0.90	9.62	1.00
P1A.R.2	1559.14	0.00	1.00	1545.33	0.89	9.44	1.00
C1.R.3	2110.71	0.00	1.00	2045.00	0.90	9.87	1.00
C2.R.3	2213.21	0.01	0.99	2133.00	0.88	9.71	0.99
C3.R.3	1877.15	0.01	1.00	1822.00	0.87	9.41	0.99
C.R.3	2067.02	0.01	1.00	2000.00	0.88	9.66	0.99
P1A1.R.3	2104.20	0.00	0.99	2021.00	0.90	9.89	1.00
P1A2.R.3	2299.02	0.01	0.99	2211.00	0.88	9.81	0.99
P1A3.R.3	2047.54	0.00	1.00	1970.00	0.90	9.87	1.00
P1A.R.3	2150.25	0.00	0.99	2067.33	0.90	9.86	1.00
